# Xenograft tumors derived from malignant pleural effusion of the patients with non-small-cell lung cancer as models to explore drug resistance

**DOI:** 10.1186/s40880-018-0284-1

**Published:** 2018-05-09

**Authors:** Yunhua Xu, Feifei Zhang, Xiaoqing Pan, Guan Wang, Lei Zhu, Jie Zhang, Danyi Wen, Shun Lu

**Affiliations:** 1 Department of Shanghai Lung Cancer Center, Shanghai Chest Hospital, Shanghai Jiao Tong University, 241 Huaihai West Road, Shanghai, 200030 China; 2 Shanghai LIDE Biotech Co., Ltd, 887 Zuchongzhi Rd, Pudong, Shanghai, 201203 China; 3 GenomiCare Biotechnology (Shanghai) Co., Ltd., Shanghai, 200233 China; 4 Department of Pathology, Shanghai Chest Hospital, Shanghai Jiao Tong University, Shanghai, 200030 China

**Keywords:** Drug resistance, Target treatment, Lung cancer, Pleural effusion, Patient-derived xenografts

## Abstract

**Background:**

Non-small cell lung cancer (NSCLC) patients with epidermal growth factor receptor (*EGFR*) mutations or anaplastic lymphoma kinase (*ALK*) fusions show dramatic responses to specific tyrosine kinase inhibitors (TKIs); however, after 10–12 months, secondary mutations arise that confer resistance. We generated a murine xenograft model using patient-derived NSCLC cells isolated from the pleural fluid of two patients with NSCLC to investigate the mechanisms of resistance against the ALK- and EGFR-targeted TKIs crizotinib and osimertinib, respectively.

**Methods:**

Genotypes of patient biopsies and xenograft tumors were determined by whole exome sequencing (WES), and patients and xenograft-bearing mice received targeted treatment (crizotinib or osimertinib) accordingly. Xenograft mice were also treated for prolonged periods to identify whether the development of drug resistance and/or treatment responses were associated with tumor size. Finally, the pathology of patients biopsies and xenograft tumors were compared histologically.

**Results:**

The histological characteristics and chemotherapy responses of xenograft tumors were similar to the actual patients. WES showed that the genotypes of the xenograft and patient tumors were similar (an echinoderm microtubule-associated protein-like 4-*ALK* (*EML4*–*ALK)* gene fusion (patient/xenograft: CTC15035_EML4–ALK_) and *EGFR* L858R and T790M mutations (patient/xenograft: CTC15063_EGFR L858R, T790M_)). After continuous crizotinib or osimertinib treatment, WES data suggested that acquired *ALK* E1210K mutation conferred crizotinib resistance in the CTC15035_EML4–ALK_ xenograft, while decreased frequencies of *EGFR* L858R and T790M mutations plus the appearance of v-RAF murine sarcoma viral oncogene homolog B (*BRAF*) G7V mutations and phosphatidylinositol-4-phosphate 3-kinase catalytic subunit type 2 alpha (*PIK3C2A*) A86fs frame shift mutations led to osimertinib resistance in the CTC15063_EGFR L858R, T790M_ xenografts.

**Conclusions:**

We successfully developed a new method of generating drug resistance xenograft models from liquid biopsies using microfluidic technology, which might be a useful tool to investigate the mechanisms of drug resistance in NSCLC.

**Electronic supplementary material:**

The online version of this article (10.1186/s40880-018-0284-1) contains supplementary material, which is available to authorized users.

## Background

Lung cancer is highly prevalent and a leading cause of cancer-related mortality worldwide [1] and in China [2]. Non-small cell lung cancer (NSCLC) accounts for approximately 85% of all new lung cancer cases [3]. Interestingly, the mortality rates of lung cancer differ significantly among developed countries [4].

*EGFR* mutations occur in up to 50% of all East Asian lung adenocarcinoma patients, while a genetic rearrangement resulting in the fusion of the 5′ region of *EML4* to the 3′ region of *ALK* occurs in 2%–5% of NSCLC patients [5, 6]. *EML4*–*ALK* fusions are most prevalent among young adenocarcinoma patients who are light or never smokers [5, 6]. Additionally, single exon 19 deletions have been described in lung adenocarcinoma [7].

Initially, first-generation TKIs such as gefitinib (for EGFR-positive NSCLC) and crizotinib (for *EML4*–*ALK* fusion-positive NSCLC) demonstrate strong anti-tumor activity; however, most patients develop resistance and subsequently relapse. In *EML4*–*ALK* fusion-positive NSCLC patients, resistance results from further mutations in *ALK*, with the L1196M mutation predominating [8]. The third-generation *ALK* TKI, lorlatinib is effective against L1196M mutant ALK, but leads to another resistance mutation, L1198F, which in turn results in a re-sensitization to crizotinib [9, 10]. Reductions in the efficacy of the EGFR TKI gefitinib are most often caused by secondary mutations in EGFR, approximately 50% of which are T790M mutations, which most often occur within 9–14 months of EGFR-TKI treatment [11].

The third-generation EGFR TKI osimertinib is effective for treating *EGFR* T790M mutant NSCLC patients with advanced disease [12], but resistance still occurs, resulting in secondary relapse [13, 14]. Previous studies have shown that the response of patient-derived xenografts to EGFR or ALK TKIs closely approximates the clinical outcomes observed in the donors’ responses to similar TKI treatments [15–17]. Thus, xenografts are useful tools to investigate mechanisms of NSCLC drug resistance, for which it is difficult to get second or third patient-derived biopsies. Ideally, NSCLC patients should undergo secondary biopsies of their primary tumors or metastases to characterize their specific drug resistance profiles. However, the implementation of secondary biopsies is often limited by the location of the lesion or metastases, patient willingness to undergo additional invasive procedures, and other factors, all of which confound efforts to improve the clinical outcomes of targeted therapy. Approximately 50% of NSCLC patients develop malignant pleural effusion. Minimally invasive drainage of accumulated pleural effusion can, therefore, reduce chest discomfort while providing pleural fluid specimens for secondary biopsy and xenograft modeling.

In this study we used a murine patient-derived xenograft model with NSCLC cells isolated from the pleural fluid of two NSCLC patients under crizotinib and osimertinib treatments, to evaluate the development of resistance mechanisms.

## Patients and methods

### Patients

Our study was approved by the Institutional Review Board of Shanghai Chest Hospital (Ethical Approval Number KS1513; 2015), and written informed consent was obtained from all patients prior to participation in our study. Patients were chosen according to the following criteria: advanced NSCLC patients with mutations, rearrangements or gene fusions and malignant pleural effusion. Pleural fluid samples were obtained from two patients (patients CTC1503_EML4–ALK_ and CTC15063_EGFR L858R, T790M_) who were diagnosed with NSCLC with malignant pleural effusion and underwent treatment at our institution.

### Establishment of xenograft models and in vivo drug treatments

Malignant tumor cells were isolated from the pleural fluid of patients using the ClearCell FX1 system (Clearbridge BioMedics Pte Ltd, Singapore) according to the manufacturer’s protocol. Tumor cells were subcutaneously inoculated into both flanks of 6–8-week-old female CB17-SCID mice (Vital River Laboratory Animal Technology Co Ltd, Beijing, China), with six mice per group. Tumors were measured twice weekly with a caliper, and tumor volumes were calculated using the formula: volume = (length × width^2^)/2. Tumors generated from malignant tumor cells isolated from patients CTC15035_EML4–ALK_ and CTC15063_EGFR L858R, T790M_ using this method are subsequently referred to as the CTC15035_EML4–ALK_ and CTC15063_EGFR L858R, T790M_ xenograft models, respectively.

**Fig. 1:**
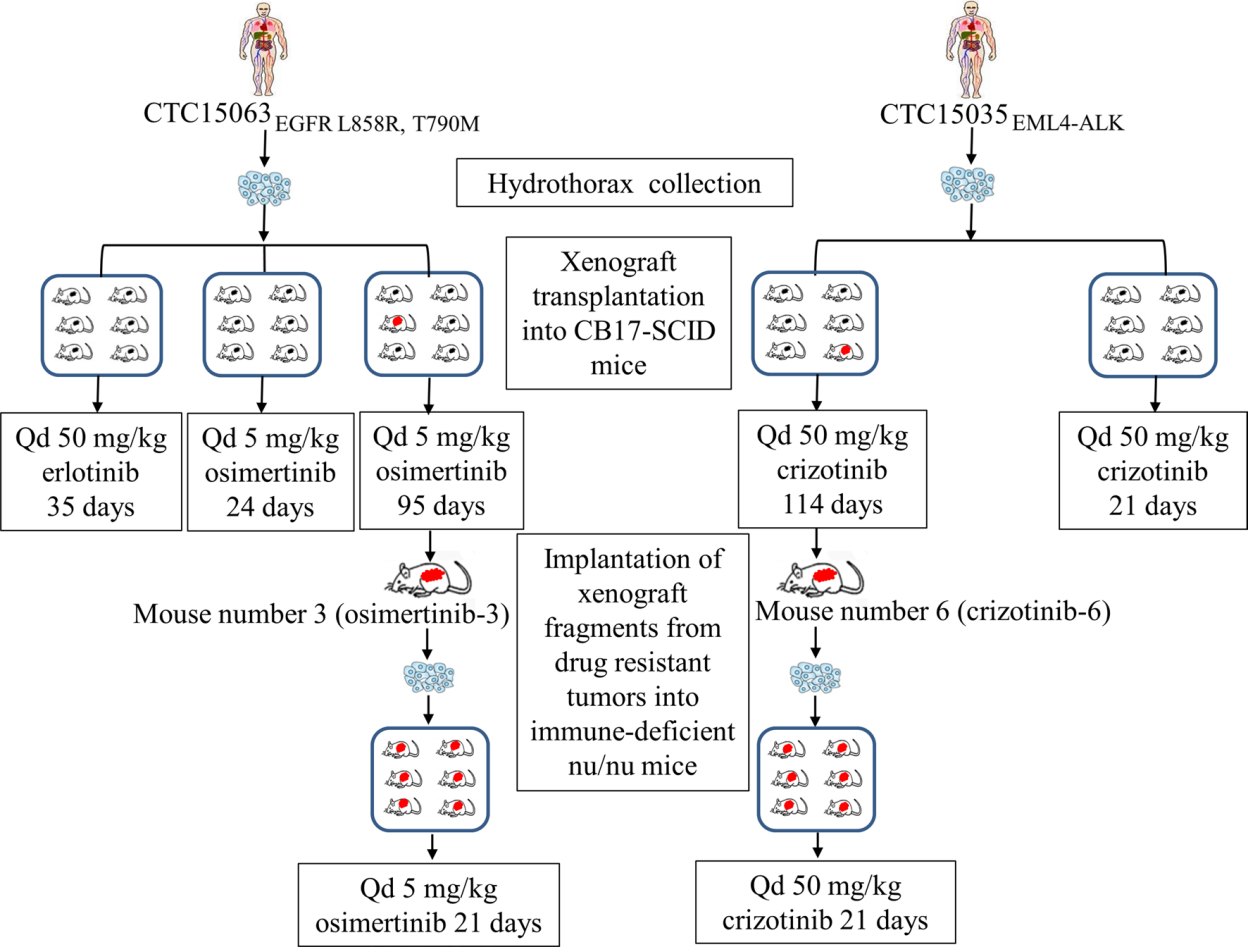
Flow chart of this study

In long-term experiments, crizotinib (114 days) and osimertinib (95 days) treatments were continued until resistance was detected based on TGI < 100% and T/C > 0 in at least one of six mice. Tumors from these resistant mice (osimertinib mouse number 3 (osimertinib-3) and crizotinib mouse number 6 (crizotinib-6) were then inoculated in the right flanks of six immune-deficient nu/nu mice (Vital River Laboratory Animal Technology Co Ltd) for further measurements (Fig. 1).

When tumors reached 100–300 mm^3^, the mice were randomly divided into three groups, with six mice of similar average tumor volume in each group. Vehicle (0.5% hydroxypropyl methylcellulose and 0.5% Tween-80) was administered orally to the control group once per day. Mice with CTC15035_EML4–ALK_ or crizotinib-6 tumors received 50 mg/kg crizotinib (SelleckChem, Houston, TX, USA) orally once per day, while mice with CTC15063_EGFR L858R, T790M_ or osimertinib-3 tumors received 50 mg/kg erlotinib (SelleckChem) or 5 mg/kg osimertinib (SelleckChem) orally once per day.

Tumor sizes were used to calculate T/C values, which served as indicators of anti-tumor efficacy: T/C = (T_ti_ − T_t0_)/(V_ci_ − V_c0_). Tumor volumes were also used to calculate tumor growth inhibition (TGI) rates according to the following formula: TGI (%) = [1 − (T_ti_ − T_t0_)/(V_ci_ − V_c0_)] × 100, where T_ti_ indicates tumor volume of the treatment group, T_t0_ indicates tumor volume of the treatment group on the first day of treatment, V_ci_ indicates tumor volume of vehicle control group, and V_c0_ indicates tumor volume of the vehicle group on the first day of treatment.

### Histology

Biopsy samples of tumor tissues and xenografts from patients CTC15035_EML4–ALK_ and CTC15063_EGFR L858R, T790M_ were fixed in 10% buffered formalin within 30 min after resection/collection. Tissues were then subjected to routine hematoxylin and eosin (H&E) staining, and NSCLC diagnoses were confirmed by a qualified pathologist.

### Whole exome sequencing (WES)

Genomic DNA from tumors and xenografts was fragmented and hybridized using the SureSelect Human All Exome kit (version 5, Agilent Technologies, Santa Clara, CA, USA). Exome sequences were enriched based on the consensus coding sequence (CCDS) data base (https://www.ncbi.nlm.nih.gov/CCDS/) using the SureSelect software (Illumina, San Diego, CA, USA), and the shotgun libraries were sequenced with a paired-end read length of 2 × 150 bases on the HiSeq Xten platform (Illumina) using the CAVSAVR, version 1.8, software (Illumina) with default parameters. Adapter sequences were removed to obtain high-quality reads. Contaminating mouse sequences in the xenograft data were removed using our proprietary bioinformatics program (xenograft tool) to improve the sensitivity and specificity of variation detection. All xenograft reads were first mapped to the murine genome using the Burrows–Wheeler Aligner with 18K-mer parameters. Using the xenograft tool, reads that aligned to murine genomic sequences were mapped with 80K-mer parameters to a set of human and mouse homologous genome regions that was constructed based on human–mouse sequence alignments of BLASTZ results. BLASTZ, an independent implementation of the Gapped BLAST algorithm was specifically designed for aligning two entire mammalian genomes [18].

The high-quality reads from resected patient samples and the xenograft reads remaining after xenograft tool filtering were aligned to the NCBI human reference genome (hg19) using Burrows–Wheeler Aligner software to identify single-nucleotide variants (SNVs).

### Identification of mutations in drug-resistant xenograft tumors

Localized insertion/deletion (InDel) mutations were analyzed with reads in FASTQ format using the Genome Analysis Toolkit (GATK), version 3.5 (https://software.broadinstitute.org/gatk/). Regions that required realignment were identified using the GATK Realigner Target Creator. For SNV detection, the MuTect algorithm was used to identify candidate SNVs in xenografts that exhibited drug resistance based on comparison to control xenografts from the same patient. The ANNOVAR software (http://annovar.openbioinformatics.org/en/latest/) was used for SNV annotation. The possible effects of nonsynonymous mutations on the encoded proteins were predicted using the dbNSFP database, version 3.1 (http://varianttools.sourceforge.net/Annotation/DbNSFP), by collating outputs from the SIFT32 and Polyphen2 prediction programs. Candidate somatic resistance InDels were identified using InDelocator (http://www.broadinstitute.org/cancer/cga/indelocator) based on comparisons to control xenografts from the same patient. Candidate InDels were only considered when they were supported by ≥ 5 reads and when the ratio of the number of supporting reads to the maximum breakpoint read depth was > 0.05. All InDel calls were manually reviewed using the Integrative Genomics Viewer (http://software.broadinstitute.org/software/igv/) before being annotated with ANNOVAR. Transcripts from gene fusions were identified according to the Ion Torrent AmpliSeq RNA Fusion Lung Cancer Research Panel protocol (Thermo Fisher Scientific, Waltham, MA, USA), which simultaneously sequences 70 different fusion transcripts and analyzes 5′ and 3′ ALK expression. The Ion Reporter software (Thermo Fisher Scientific) was used to detect gene fusion events, indicate fusion partners, and determine fusion junctions.

### Confirmation of mutations by Sanger sequencing

Genomic DNA sequences containing the somatic mutations of drug-resistant xenograft were amplified by touchdown PCR using primers that targeted the variant sequences with thermal cycling performed at 98 °C for 10 min; 94 °C for 2 min; 10 cycles of 94 °C for 10 s, 75–50 °C for 70 s (reduced 2.5 °C every cycle), 72 °C for 45 s; 20 cycles of 94 °C for 15 s, 50 °C for 30 s, 74 °C for 45 s; and finally 72 °C for 150 s. Sizes of the amplified fragments were confirmed by agarose gel electrophoresis, and the fragments were purified using Agencourt Ampure XP beads (Beckman Coulter, Brea, CA, USA).

### Gene functional annotation

Functional annotation was performed using the DAVID Bioinformatics Resources, version 6.8 (https://david.ncifcrf.gov/content.jsp). The interrogating dataset contained the mutated genes, and the background dataset consisted of all genes in the human genome. Genes annotated in the Kyoto Encyclopedia of Genes and Genomes (KEGG; http://www.kegg.jp/) or Gene Ontology (GO; http://www.geneontology.org/) databases as functioning in signaling pathways, biological processes, or molecular functions were subjected to Fisher’s exact test. Enrichment was considered statistically significant at *P* < 0.05.

## Results

### Morphology and pathology of resected patient and xenograft tumors

#### Patient CTC15035_EML4–ALK_

**Fig. 2:**
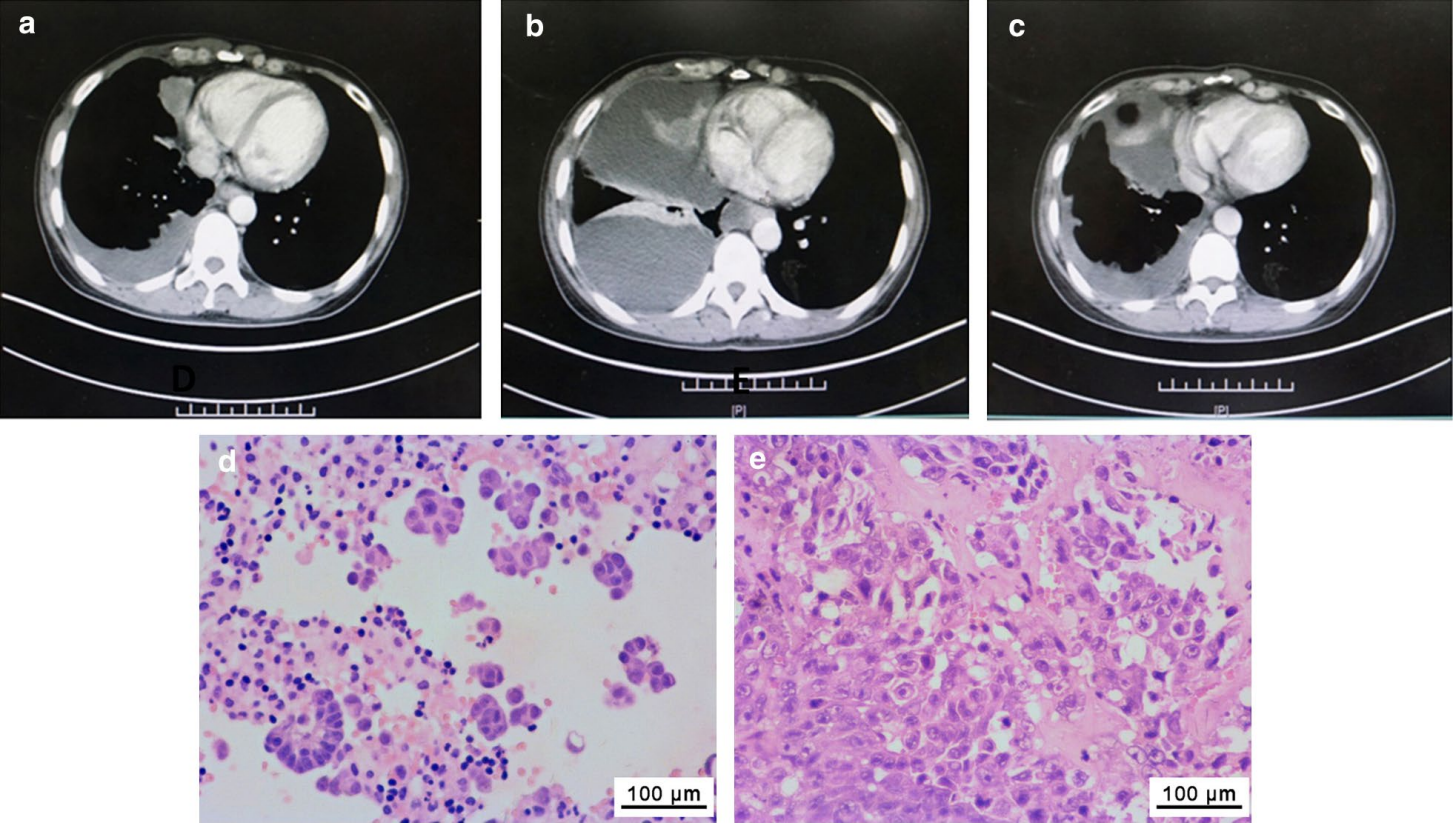
Computed tomography (CT) scans and pathology for patient CTC15035_EML4–ALK_ with stage IV bronchogenic carcinoma and the patient-derived xenograft tumors. **a** Baseline CT chest scan on 2014-11-14 showed there was a nodule in the middle lobe of the right lung and a small amount pleural effusion in the lower lobe of the right lung. **b** After two gemcitabine plus carboplatin chemotherapy cycles, CT of disease progression on 2014-12-23 showed the pleural effusion increased significantly compared with that of baseline. **c** After 2 months of crizotinib targeted therapy, CT chest scan on 2015-3-12 showed the disease was stable and the pleural effusion decreased compared with that of pre-crizotinib treatment. Pathology was examined by H&E staining of the tumor biopsy from **d** patient CTC15035_EML4–ALK_ when pleural effusion increased significantly and, **e** a xenograft tumor from CTC15035_EML4–ALK_. Histology of xenograft tumors was well-matched with those of primary tumors

CTC15035_EML4–ALK_ tumor cells were obtained from a 47-year-old female patient (patient CTC15035_EML4–ALK_) who presented with stage IV lung adenocarcinoma with pleura, retroperitoneal lymph node, and brain metastases (Fig. 2a). She was initially treated with two cycles of gemcitabine plus carboplatin. However, disease progression occurred with malignant pleural effusion and mass enlargement (Fig. 2b), at which point pleural fluid was collected to generate the CTC15035_EML4–ALK_ xenograft tumors. A tumor biopsy sample collected from the patient was ALK fusion-positive and crizotinib was subsequently applied [19, 20], which stabilized the patient’s disease for 18 months (Fig. 2c). Prior to crizotinib treatment, the histological characteristics of the tumor biopsy (Fig. 2d) were highly similar to the xenograft tumors (Fig. 2e).

#### Patient CTC15063_EGFR L858R, T790M_

**Fig. 3:**
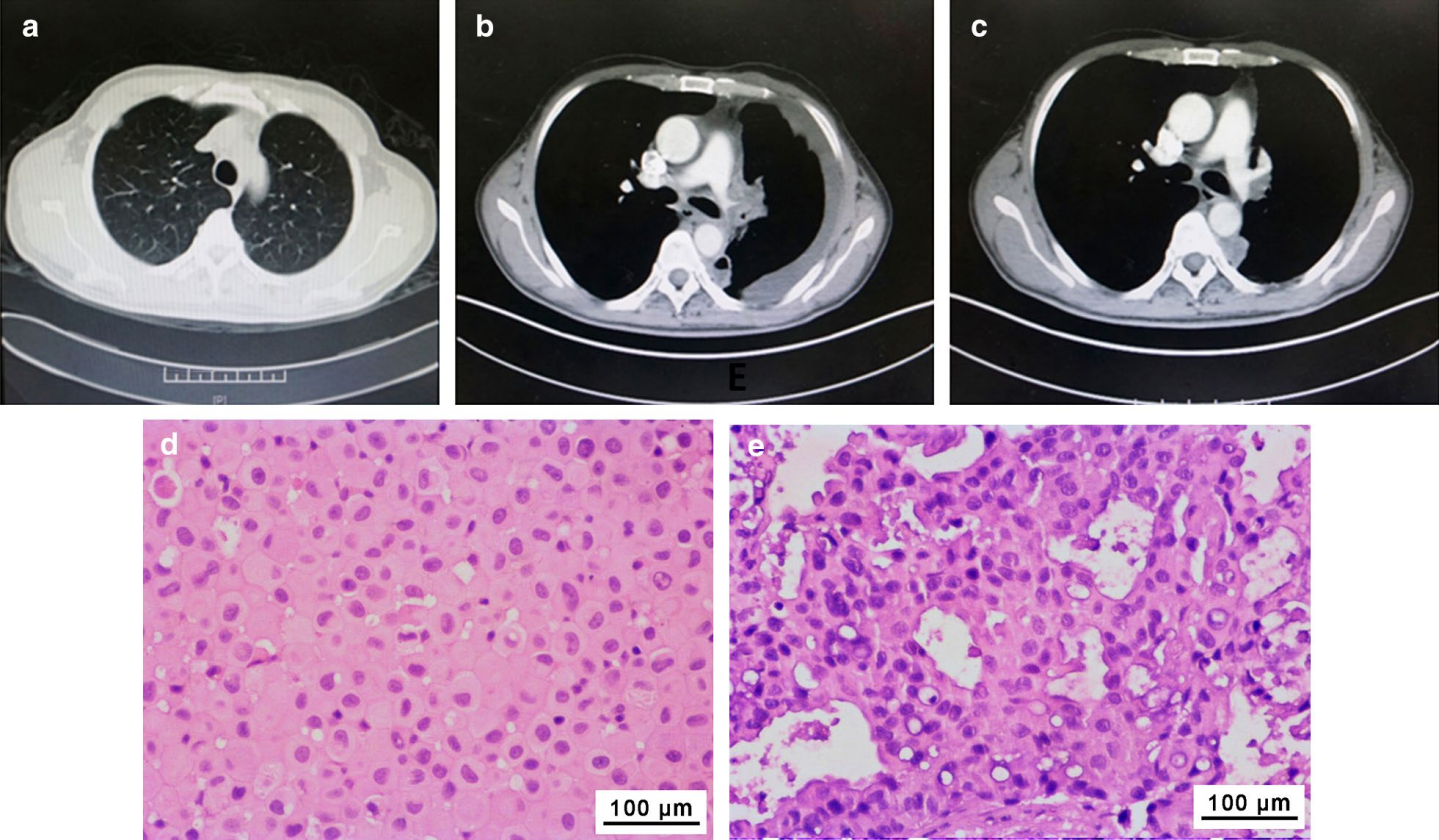
Computed tomography (CT) scans and pathology for patient CTC15063_EGFR L858R, T790M_ and the patient-derived xenograft tumors. CT chest scans of patient CTC15063_EGFR L858R, T790M_ were recorded 4.5 years after undergoing surgical resection and subsequent adjuvant chemotherapy (navelbine plus cisplatin). **a** Disease recurrence was found in a CT scan as a right sub-pleural nodule on 2012-3-12; **b** disease progression 26 months after erlotinib treatment was visible in a CT scan as malignant pleural effusion on 2014-6-27; **c** CT reexamination after 2 months of osimertinib treatment showed stable disease on 2015-10-8. Pathology (H&E staining) of tumor biopsy from **d** patient CTC15063_EGFR L858R, T790M_ and **e** a xenograft CTC15063_EGFR L858R, T790M_ tumor after osimertinib treatment

CTC15063_EGFR L858R, T790M_ tumor cells were obtained from a 52-year-old male NSCLC patient (patient CTC15063_EGFR L858R, T790M_), who had previously presented with stage IV lung cancer with pleura, peritoneum, and right lung metastases. The patient underwent surgical tumor resection and adjuvant chemotherapy with navelbine plus cisplatin. At a follow-up visit approximately 4.5 years later, disease recurrence with right pleura metastasis was found (Fig. 3a). A tumor biopsy specimen tested positive for the *EGFR* L858R mutation, so the patient was treated with erlotinib. A partial response was achieved, but disease progression with malignant pleural effusion occurred 26 months after beginning erlotinib (Fig. 3b). Pleural fluid was then collected, and malignant cells isolated from the pleural fluid were used to generate the CTC15063_EGFR L858R, T790M_ xenografts. Subsequently, another biopsy sample of the tumor tested positive for *EGFR* L858R and T790M mutations, so the patient was treated with osimertinib, and his disease was stabilized for 13 months (Fig. 3c). Histological characteristics of the CTC15063_EGFR L858R, T790M_ tumor xenografts were highly similar to the tumor biopsy (Fig. 3d, e).

### The development of crizotinib resistance in *EML4*–*ALK* NSCLC and xenografts was related to an E1210K mutation in the ribose-binding pocket of *ALK*

**Fig. 4:**
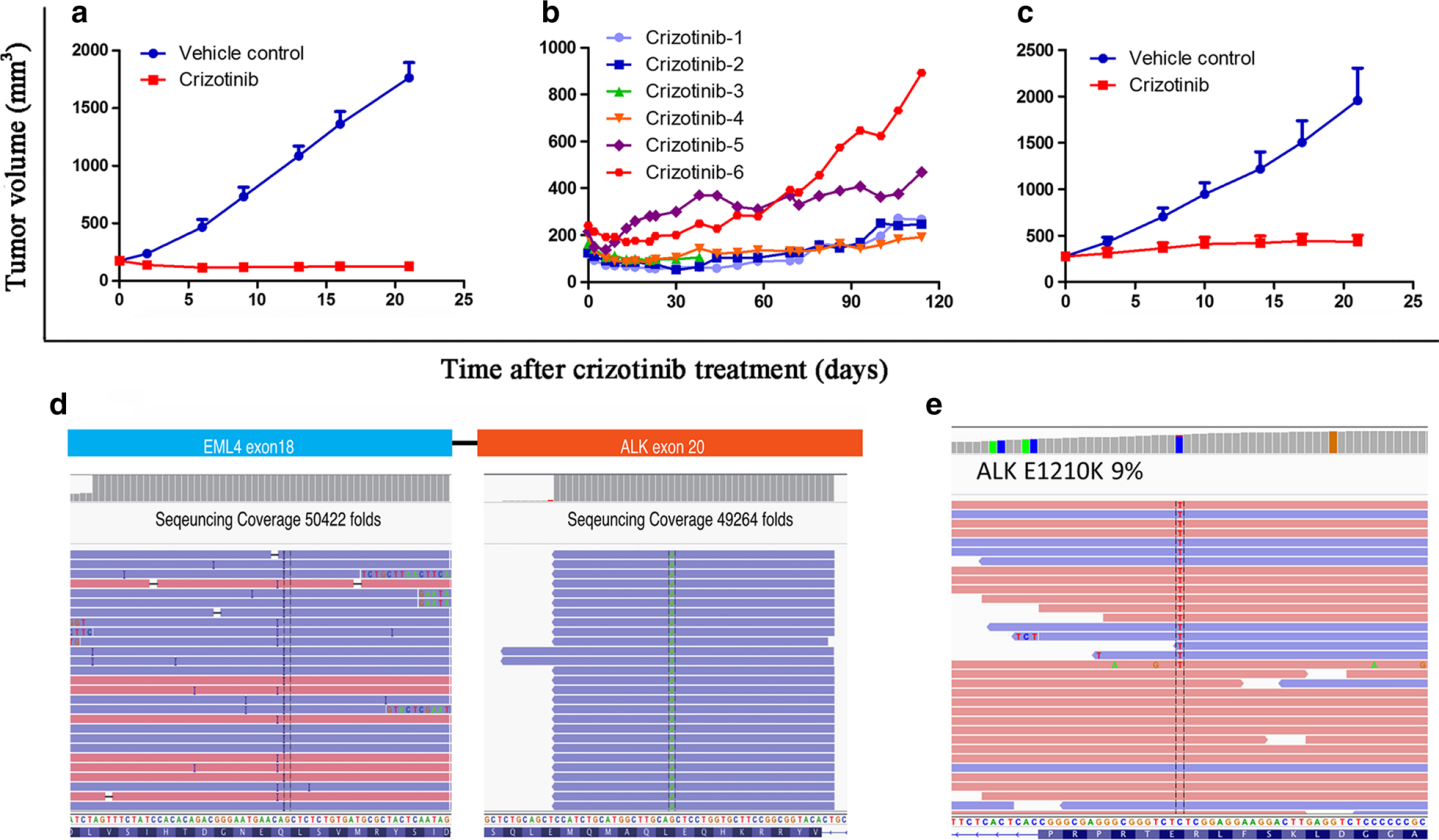
The effect of crizotinib on CTC15035_EML4–ALK_ xenografts and their development of crizotinib resistance. **a** CTC15035_EML4–ALK_ xenograft growth curves for female nu/nu mice (mean ± SEM, n = 6) treated with 50 mg/kg crizotinib or vehicle control for 21 days. **b** CTC15035_EML4–ALK_ xenograft growth curves in six female nu/nu mice (crizotinib 1–6) for 114 days with 50 mg/kg crizotinib (mean ± SEM, n = 6). **c** Secondary CTC15035_EML4–ALK_ xenografts derived from crizotinib-6 under 50 mg/kg crizotinib or vehicle control treatments (mean ± SEM, n = 6). **d** Fusion transcripts of *EML4* Exon 18 to *ALK* Exon 20, which occurred in the tumor biopsy from patient CTC15035_EML4–ALK_ and the CTC15035_EML4–ALK_ xenografts, were identified by RNA amplification sequencing with an ultra-deep sequencing depth of approximately 50,000-fold of the *EML4*–*ALK* fusion locus; **e** the novel acquired *ALK* E1210K mutation in crizotinib-6 xenografts at a frequency of 9% (**d**, **e** are displayed with Integrative Genomics Viewer)

Patient CTC15035_EML4–ALK_ and mice with the tumors derived from CTC15035_EML4–ALK_ were treated with crizotinib. The effects of crizotinib treatment on xenograft tumor sizes are presented in Fig. 4a. On day 21, the mean xenograft tumor volume of the CTC15035_EML4–ALK_ control group grew from 176.65 ± 24.77 to 1764.72 ± 34.43 mm^3^, whereas under 50 mg/kg crizotinib treatment the xenograft tumors shrank from 176.18 ± 20.00 to 128.26 ± 34.43 mm^3^ (*P *< 0.001), with a TGI% of 103.2% and a T/C% of − 3.02% on day 21 (Fig. 4a).

On day 114 of crizotinib treatment, the tumor in the crizotinib-6 mouse had grown to 892.98 mm^3^, which was significantly larger than tumors in the other mice in the crizotinib treatment group (Fig. 4b). To further evaluate the crizotinib-6 tumor, we generated new xenografts by implanting fragments of the crizotinib-6 tumor into the flanks of naive nu/nu mice. These secondary xenografts were treated with 50 mg/kg crizotinib once per day or vehicle control for 21 days (Fig. 4c). The control group tumors grew from 278.15 ± 31.52 to 1954.75 ± 347.77 mm^3^, whereas under 50 mg/kg crizotinib treatment, the xenograft tumors grew from 273.86 ± 36.55 to 432.29 ± 71.20 mm^3^ (*P *< 0.001), with a TGI% of 90.55% and a T/C% of 9.45% on day 21. Unlike the previous experiment in which all CTC15035_EML4–ALK_ tumors shrank following crizotinib treatment, these secondary crizotinib-6 tumors exhibited a slow rate of growth despite crizotinib treatment (Fig. 4c), indicating acquired resistance had been achieved.

Genomic DNA from tumor tissues of patient CTC15035_EML4–ALK_ and those of crizotinib-6 were subjected to WES. These deep sequencing results are summarized in Additional file 1: Table S1, and the genetic variations identified in the samples are summarized in Table 1. The transcript fusion of *EML4* exon 18 to *ALK* exon 20 was identified in both the patient’s tumor and crizotinib-6 xenografts (Fig. 4d). A novel acquired *ALK* mutation, E1210K, which mapped to the ribose-binding pocket of *ALK*, was also identified in crizotinib-6 at a frequency of 9% (Fig. 4e). Although the E1210K mutation has not been biochemically characterized, a previous study reported that the E1210K mutation conferred crizotinib resistance in vitro [21].

**Table 1:** Whole exome sequencing of patients-derived biopsies and xenografts

Sample	Yield (Gbases)	Reads (× 10^6^)	% ≥ Q30 bases (PF)	Key mutations
Biopsy from patient CTC15035_EML4–ALK_	13.63	90.88	90.73	*EML4*–*ALK* fusion (EML4 exon18–ALK exon 20)
Crizotinib-6 xenograft	27.77	185.10	91.38	*EML4*–*ALK* fusion (EML4 exon18–ALK exon 20); *ALK*: E1210K (9%)
Biopsy from patient CTC15063_EGFR L858R, T790M_	14.72	98.15	90.94	*EGFR*: L858R (85.7%) and T790M (71.5%)
Xenograft from patient CTC15063_EGFR L858R, T790M_	25.09	167.24	90.04	*EGFR*: L858R (83.3%) and T790M (77.6%)
Osimertinib-3 xenograft	27.38	182.52	90.73	*EGFR*: L858R (53.6%) and T790M (41.7%); *PIK3C2A*: R86fs (11%); *BRAF*: G7V (11.5%)

*Gbases* gigabases, *M* million, *PF* post filter, *EML4*–*ALK* echinoderm microtubule-associated protein-like 4–anaplastic lymphoma kinase, *EGFR* epidermal growth factor receptor

### The development of osimertinib resistance in EGFR L858R, T790M NSCLC and xenografts was related to secondary mutations in *BRAF* and *PIK3C2A* combined with reduced *EGFR*-T790M mutations

**Fig. 5:**
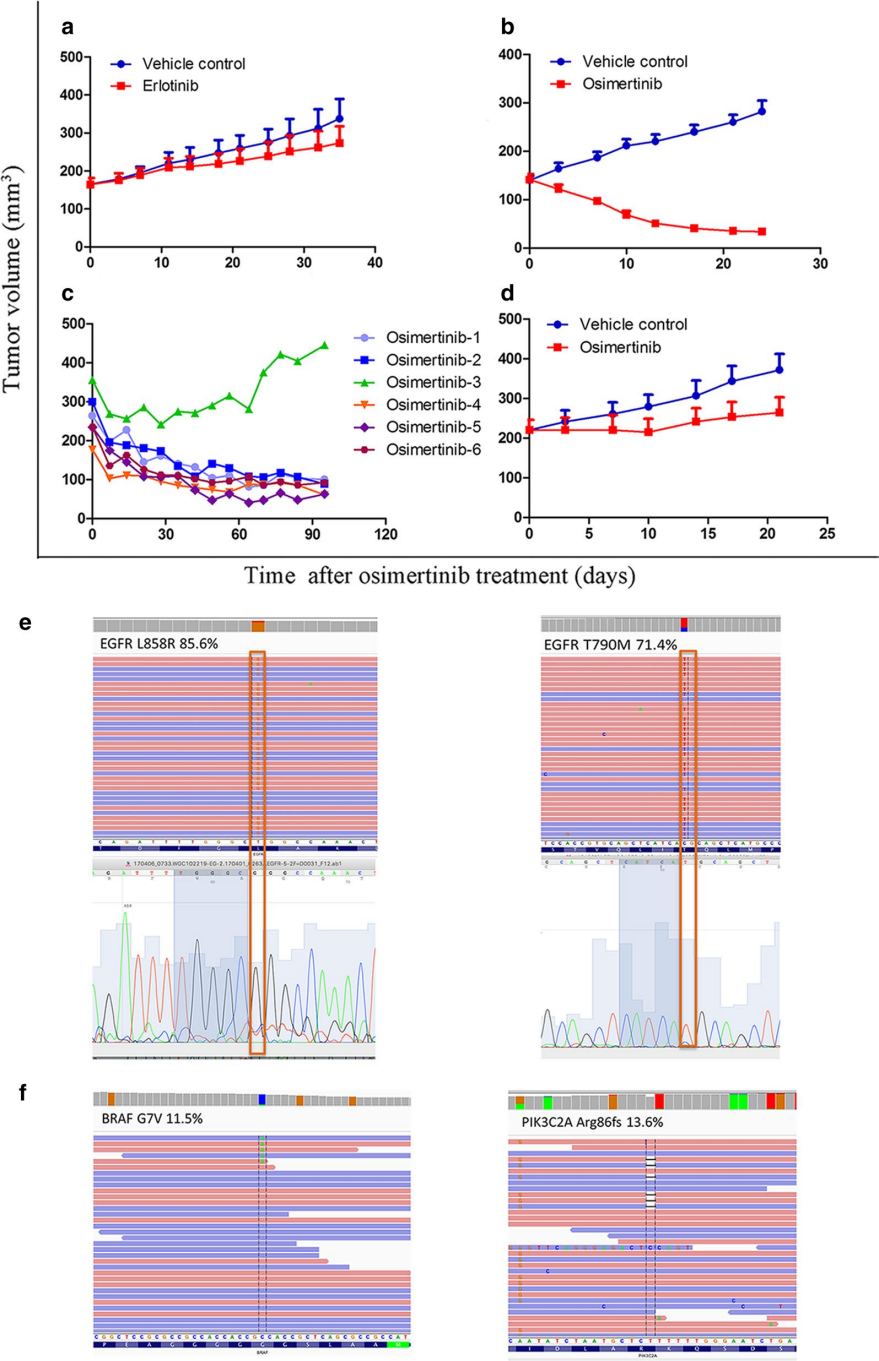
The effect of osimertinib on CTC15063_EGFR L858R, T790M_ xenografts tumor inhibition or tumor re-development due to osimertinib resistance. **a** CTC15063_EGFR L858R, T790M_ xenograft growth curves for female nu/nu mice (mean ± SEM, n = 6) treated with 50 mg/kg erlotinib or vehicle control for 35 days. **b** CTC15063_EGFR L858R, T790M_ xenograft growth curves for female nu/nu mice (mean ± SEM, n = 6) treated with 5 mg/kg osimertinib or vehicle for 24 days. **c** CTC15063_EGFR L858R, T790M_ xenograft growth curves of six female nu/nu mice (osimertinib 1–6) treated with 5 mg/kg osimertinib for 95 days. **d** Secondary CTC15063_EGFR L858R_ xenografts derived from osimertinib-3 under 5 mg/kg osimertinib or vehicle control treatments for 21 days (mean ± SEM, n = 6). **e** Frequencies of the *EGFR* L858R and T790M mutations in the tumor biopsy from patient CTC15063_EGFR L858R, T790M_ (85.6% and 71.6%, respectively) were similar to those in the initial CTC15063_EGFR L858R, T790M_ xenograft tumors (83.3% and 77.6%, respectively), but were higher than those in osimertinib-3-derived secondary xenografts (53.6% and 41.7%, respectively); **f** 33 novel secondary mutations were identified in osimertinib-3-derived xenografts, including *BRAF* (G7V) (11.54%) and *PIK3C2A* (A86fs) (13.64%)

Mice with tumors derived from CTC15063_EGFR L858R, T790M_ were treated with erlotinib to confirm the partial response previously observed in the patient. Volumes of CTC15063_EGFR L858R, T790M_ xenografts were not reduced by erlotinib treatment. The mean size of control xenografts on days 0 and 35 were 164.82 ± 16.68 and 337.7 ± 52.15 mm^3^, respectively, and for 5 mg/kg-treated tumors they were 273.35 ± 43.94 mm^3^ on day 35 vs. 164.37 ± 17.21 mm^3^ on day 0 (*P *< 0.001), with a TGI% of 36.96% and a T/C% of 63.04% (Fig. 5a). Patient CTC15063_EGFR L858R, T790M_ and the mice with xenograft tumors derived from him were treated with osimertinib. The effects of osimertinib treatment on xenograft tumor volumes are presented in Fig. 5b. On day 21, the mean tumor volume for the control group was significantly greater than that of the osimertinib treatment group (282.59 ± 22.02 mm^3^ vs. 34.15 ± 4.27 mm^3^, TGI% = 174.98%, T/C% = − 74.98% (*P *< 0.001), whereas they were not different at day 0 (139.88 ± 9.40 mm^3^ vs. 141.15 ± 11.9 mm^3^), demonstrating a significant initial anti-tumor response. Osimertinib treatment was continued, and the osimertinib-3 tumor reached 445.16 mm^3^ on day 95 (Fig. 5c).

To further evaluate the osimertinib-3 tumor, we generated new xenografts in naive nu/nu mice using fragments from the osimertinib-3 tumor. These secondary xenografts were treated with 5 mg/kg osimertinib once per day or vehicle control for 21 days (Fig. 5d). The initial xenograft sizes were 220.73 ± 25.60 and 220.65 ± 25.51 mm^3^ for control and osimertinib-treated mice, respectively. After 21 days, the tumors grew to 372.39 ± 40.18 and 265.41 ± 37.97 mm^3^ (P < 0.001), respectively, with a TGI% of 70.49% and a T/C% of 29.51% for the osimertinib-treated xenografts (Fig. 5d). Unlike the previous experiment in which all CTC15063_EGFR L858R, T790M_ tumors shrank following osimertinib treatment, these secondary osimertinib-3 tumors exhibited a slow rate of growth despite osimertinib treatment, indicating the achievement of acquired resistance. WES showed that the tumor from patient CTC15063_EGFR L858R, T790M_, the osimertinib-sensitive CTC15063_EGFR L858R, T790M_ xenograft tumors, and the osimertinib-resistant osimertinib-3 tumor had the *EGFR* mutations, L858R and T790M. As shown in Table 1, the frequencies of the L858R and T790M mutations in osimertinib-3 (53.6% and 41.7%, respectively) were lower (*P *= 0.0029 vs. L858R, and *P *< 0.0001 vs. T790M) than those in the osimertinib-sensitive CTC15063_EGFR L858R, T790M_ xenografts (83.3% and 77.6%, respectively) or from patient-derived CTC15063_EGFR L858R, T790M_ tissue (85.6% and 71.4%, respectively; Fig. 5e). Novel secondary mutations in *BRAF* (G7V) and *PIK3C2A* (A86fs) were found only in osimertinib-3 xenografts, occurring at rates of 11.54% and 13.64%, respectively (Fig. 5f; Table 1). Previous studies have shown that genetic variants of *BRAF* and *PIK3C2A* are associated with clinical outcome in NSCLC patients [22–24]. Therefore, our results suggested that osimertinib resistance had been acquired through a combination of secondary mutations in *BRAF* and *PIK3C2A* and reduced frequency of the *EGFR* L858R and T790M mutations.

## Discussion

The histological characteristics, genotypes, and chemotherapy responses of our patient-derived xenograft tumors were similar to those of the patients’ primary tumor samples. The tumor from patient CTC15035_EML4–ALK_ and the CTC15035_EML4–ALK_ xenografts generated from it contained the *EML4*–*ALK* gene fusion, and the tumor from patient CTC15063_EGFR L858R, T790M_ and the CTC15063_EGFR L858R, T790M_ xenografts generated from it contained the EGFR mutations, L858R and T790M. These results validate the use of xenograft tumors to investigate the molecular basis of TKI resistance in NSCLC.

ALK is a transmembrane receptor tyrosine kinase of the insulin receptor superfamily [25]. Various chromosomal rearrangements result in ALK fusions with oncogenic activity in NSCLC, with nearly 20 different ALK-fusion proteins described in the literature, including *EML4*–*ALK*, the most common ALK fusion in NSCLC [26]. Downstream signaling from ALK fusion proteins involve the Janus kinase/signal transducer and activator of transcription (JAK/STAT) cell survival pathway and the mitogen-activated protein kinases/extracellular signal-regulated kinases (MEK/ERK) cell proliferation pathway. *EML4*–*ALK* fusions occur in approximately 2%–5% of NSCLC cases, the majority of which are adenocarcinomas [5, 6]. Mutations within the *ALK* tyrosine kinase domain and amplification of *ALK* fusions contribute to relapse in approximately 33% of NSCLC cases undergoing TKI treatment [21, 27]. Certain SNVs in ALK occur in response to ALK-TKI treatment, including L1196M for crizotinib, G1202R and compound ALK mutations for ceritinib, and G1202R for alectinib [28–30]. A previous study showed that *ALK* E1210K conferred resistance to crizotinib in vitro [21], and our whole exome sequencing analysis revealed that the *ALK* E1210K mutation occurred in the crizotinib-resistant xenograft tumor, crizotinib-6. However, the E1210K mutation rate crizotinib-6 was relatively low (9%), which likely contributed to the partial sensitivity of crizotinib-6 to crizotinib, which was reflected by the slow rate of tumor growth in the treatment group (Fig. 4c).

Approximately 70% of NSCLC patients have tumors with *EGFR* mutations that are sensitive to EGFR-targeted TKIs that inhibit downstream signaling events by binding to the intracellular domain of EGFR [31–33]. Approximately 50% of patients with EGFR-positive tumors acquire resistance to the first- or second-generation TKIs, erlotinib, gefitinib and afatinib, through a number of mechanisms, including secondary mutations in *EGFR*, such as T790M, C797S, and L792F/Y/H and the activation of alternative signaling pathways, including hepatocyte growth factor (HGF), hepatocyte growth factor receptor (HGFR), human epidermal growth factor receptor 2 (HER2), AXL receptor tyrosine kinase (AXL), Hedgehog (Hh), insulin-like growth factor 1 receptor (IGF-1R)-mediated signaling, or perturbations in downstream proteins, such as protein kinase b (AKT) and phosphatase and tensin homolog (PTEN) signaling; additionally, inhibition of EGFR-TKI-mediated apoptosis by Bcl-2-like protein 11 (BIM) deletions have also been reported [13, 34–37]. Therefore, despite the initial benefits of these first-line TKI treatments [38–40], approximately 50% of NSCLC patients will develop resistance 9–14 months after beginning treatment [38, 41–43]. The second-generation TKIs, afatinib and dacomitinib, have demonstrated limited efficacy for mitigating secondary mutation-induced TKI resistance [44–46]. The third-generation TKI, osimertinib, irreversibly inhibits the tyrosine kinase activity induced by activating EGFR mutations and T790M-mediated resistance mutations without adversely affecting wild-type EGFR [47, 48].

We sought to clarify the mechanisms of acquired resistance to third-generation EGFR-TKIs by inducing resistance in the CTC15063_EGFR L858R, T790M_ xenografts through continuous osimertinib treatment. Neither C797S nor L792F/Y/H mutations were induced in these CTC15063_EGFR L858R, T790M_ xenograft tumors. Amplification of relevant genes, including *AKT1*, Kirsten rat sarcoma (*KRAS*), and proto-oncogene tyrosine-protein kinase Yes (*YES1*), were detected (data not shown) but were not enriched in the osimertinib-resistant xenograft tumor, osimertinib-3. The allele frequencies of L858R and T790M in tumor tissues from patient CTC15063_EGFR L858R, T790M_ and the osimertinib-sensitive CTC15063_EGFR L858R, T790M_ xenograft tumor were similar, whereas the frequencies of these mutations were significantly lower in the osimertinib-resistant xenograft tumor clone, osimertinib-3 (Table 1). These results suggested that a decrease in the L858R and T790M mutation rates contributed to acquired osimertinib resistance in osimertinib-3.

Novel secondary mutations in BRAF (G7V) and PIK3C2A (A86fs) only occurred in osimertinib-3 (Fig. 5f). Mutations in BRAF occur in approximately 3% of NSCLC cases [49]. A recent study by Ichihara et al. [50] showed that Src family kinases are involved in sustaining MAPK signaling in EGFR-TKI-sensitive lung cancer cells treated with osimertinib, and that mutations in PIK3C2A and phosphatidylinositol 4,5-bisphosphate 3-kinase catalytic subunit beta (PIK3CB) attenuated the anti-tumor effects of osimertinib in T790M-positive lung cancer tumors. Ichihara et al. [50] also found that combined Src family kinase inhibitor plus osimertinib treatment was effective at inhibiting the growth of osimertinib-resistant lung cancer [50]. BRAF and ERK2 are components of the MAPK pathway [51, 52]. Together, the occurrence of BRAF and PIK3C2A mutations in the osimertinib-resistant xenograft tumor and the findings of Ichihara et al. [50] suggest that under continuous EGFR-TKI treatment, MAPK signaling independently contributes to tumor cell proliferation and survival through a mechanism downstream of EGFR, which overrides the effect of EGFR-TKI treatment.

Finally, Src-mediated MAPK signaling mitigates the anti-tumor activity of osimertinib in EGFR-TKI-sensitive lung cancer, and PIK3C2A mutations attenuate the effects of osimertinib in T790M-positive lung cancer. Our findings suggest that under continuous EGFR-TKI treatment, MAPK signaling might also contribute to TKI resistance in ALK fusion-positive NSCLC via an ERK2-mediated mechanism.

One potential shortcoming of our experimental approach was that we did not obtain additional secondary biopsies from either patient when they eventually developed resistance to crizotinib or osimertinib. These samples would have provided further information regarding the mechanisms of drug resistance in NSCLC patients. Additionally, a larger cohort study will be required to assess associations with drug responses in the future.

## Conclusions

Using novel microfluidic technology, we successfully developed a new method of generating drug-resistant xenograft models from liquid biopsies. Such drug-resistant xenograft models are feasible tools to understand the mechanisms of NSCLC drug resistance.

## Additional file

**Additional file 1: Table S1.** Summary of whole exome sequencing data quality control for patient-derived biopsies and xenografts (paired-end read length 2 × 150 bp for all samples).

## Abbreviations

AKT1: RAC-alpha serine/threonine-protein kinase
AXL: AXL receptor tyrosine kinase
BCL2: B-cell lymphoma-2
BIM: Bcl-2-like protein 11
BRAF: v-Raf murine sarcoma viral oncogene homolog B
BWA: Burrows–Wheeler Aligner
EML4–ALK: echinoderm microtubule-associated protein-like 4–*ALK*
GATK: Genome Analysis Toolkit
GF-1R: insulin-like growth factor 1 receptor
GO: Gene Ontology
HER2: human epidermal growth factor receptor 2
HGF: hepatocyte growth factor
Hh: Hedgehog
H&E: hematoxylin and eosin
JAK/STAT: Janus kinase/signal transducer and activator of transcription
KEGG: Kyoto Encyclopedia of Genes and Genomes
KRAS: Kirsten rat sarcoma
MAPK: mitogen-activated protein kinase
MEK/ERK: mitogen-activated protein kinases, extracellular signal-regulated kinases
NSCLC: non-small-cell lung cancer
PCR: polymerase chain reaction
PIK3C2A: phosphatidylinositol-4-phosphate 3-kinase catalytic subunit type 2 alpha
PTEN: phosphatase and tensin homolog
Qd: one time per day
SFK: Src family kinase
SNV: single-nucleotide variant
TGI: tumor growth inhibition
TKI: tyrosine kinase inhibitor
YES1: tyrosine-protein kinase Yes
