# Supplementary material for: Xenograft tumors derived from malignant pleural effusion of the patients with non-small-cell lung cancer as models to explore drug resistance
Source: Cancer Commun (Lond). 2018 May 9;38:19. doi: 10.1186/s40880-018-0284-1 (PMC5993147; doi:10.1186/s40880-018-0284-1)
Supplement: Supplementary file 1 — Additional file 1: Table S1. Summary of whole exome sequencing data quality control for patient-derived biopsies and xenografts (paired-end read length 2 × 150 bp for all samples). [file 40880_2018_284_MOESM1_ESM.docx]

**Table S1.** Summary of whole exome sequencing data quality control for patient-derived biopsies and xenografts (paired-end read length 2×150 bp for all samples)

| Sample | Patient CTC15035 | Crizotinib-6 | Patient CTC15063 | CTC15063 | Osimertinib-3 |
| --- | --- | --- | --- | --- | --- |
| Sample Type | Tumor | xenograft | Tumor | xenograft | xenograft |
| xenograft Human DNA Percentage | 0% | 31.4% | 0% | 36.5% | 23.2% |
| Total effective data yield (Gb) | 13.6 | 8.6 | 14.7 | 9.1 | 6.0 |
| Total reads number (M) | 90.9 | 57.5 | 98.1 | 60.4 | 40.2 |
| Reads mapping rate | 99.4% | 99.1% | 99.8% | 99.0% | 96.1% |
| Properly paired mapping reads rate | 99.4% | 99.1% | 99.8% | 99.9% | 96.0% |
| No-mismatch mapping reads rate | 60.7% | 58.9% | 59.3% | 57.4% | 57.7% |
| Mismatch alignment bases rate | 0.56% | 0.62% | 0.58% | 0.71% | 0.63% |
| Capture efficiency rate on target regions | 85.1% | 53.0% | 87.7% | 57.1% | 51.2% |
| Mean depth of sequencing on official target (reads) | 168.74 | 62.44 | 187.33 | 69.76 | 42.3 |
| Fraction of official target covered | 99.8% | 83.8% | 99.9% | 85.0% | 83.5% |
| Fraction of official target covered with at least 4 reads | 99.7% | 74.0% | 99.7% | 74.9% | 71.7% |
| Fraction of official target covered with at least 10 reads | 99.2% | 62.1% | 99.0% | 63.8% | 57.8% |
| Fraction of official target covered with at least 20 reads | 96.9% | 49.5% | 96.1% | 53.6% | 45.1% |
| Fraction of official target covered with at least 60 reads | 74.7% | 26.9% | 74.2% | 32.6% | 21.4% |
| Fraction of official target covered with at least 100 reads | 53.6% | 17.5% | 54.8% | 21.5% | 11.8% |
| PCR duplication rate | 12.2% | 16.8% | 21.9% | 28.3% | 19.0% |
